# Draft Genome Sequence of Mercury-Resistant Pseudomonas putida Strain DRA525

**DOI:** 10.1128/genomeA.00370-18

**Published:** 2018-05-03

**Authors:** Kevin Drace, Stephanie Giangiuli, Claire LeSar, Adam M. Kiefer

**Affiliations:** a Department of Biology, Birmingham-Southern College, Birmingham, Alabama, USA; b Department of Chemistry, Mercer University, Macon, Georgia, USA

## Abstract

We report here the draft genome sequence of Pseudomonas putida strain DRA525, isolated from mercury-contaminated soil. This strain shows resistance to mercury and multiple antibiotics, and its genome sequence contains several gene sets known to confer resistance to heavy metals enzymatically and through multidrug efflux pumps.

## GENOME ANNOUNCEMENT

Pseudomonas putida is generally associated with the soil environment and has been extensively studied due to its ability to tolerate and/or metabolize a variety of environmental contaminants, including some heavy metals (1, 2). P. putida strain DRA525 was isolated from soil sampled near an artisanal gold mining camp in Manica Province, Mozambique, where mercury is commonly used in the gold mining process. This strain was selected for further investigation because of its resistance to HgCl_2_ (100 µM) and multiple antibiotics, a commonly reported correlation (3–5). The draft genome sequence of P. putida strain DRA525 has the potential to provide information useful in addressing questions related to the correlation of heavy metal and antibiotic resistance and the contributions to the mercury cycle made by similar bacteria in highly contaminated environments.

The draft genome sequence of P. putida DRA525 was generated by ACGT, Inc., (Wheeling, IL) using the Illumina MiSeq platform derived from combined paired-end and mate-paired libraries of 3,105,539 and 5,194,923 total read pairs, respectively. Adaptors were trimmed and short reads filtered using Trim Galore version 0.3.7 (6) and Sickle version 1.33 (7). Trimmed and filtered reads were assembled using SPAdes version 3.5 (8) into a single closed contig sequence of 6,267,599 bases, with a G+C content of 63%. The draft assembly was annotated using the Rapid Annotations with Subsystems Technology (RAST) server (9), which identified 5,571 open reading frames, and 78 tRNAs were identified using tRNAscan-SE 2.0 (10).

The average nucleotide identity (ANI) was calculated through one-way ANI and two-way ANI between P. putida DRA525 and five P. putida strains, as previously described (11). The ANI values range from 86.8% (strain W619) to 96.1% (strain S16), with values of 89.6% (strain F1), 89.8% (strain KT2440), and 95.3% (strain HB3267) for the remaining comparisons. As expected, we confirmed the presence of the mercuric resistance (*mer*) operon (*merD*, *merA*, *merC*, *merP*, *merT*, and *merR*) in this strain. In addition, two copies of a gene set containing *arsH*, *acr3*, *arsC*, and *arsR* suggest arsenic resistance (12, 13), and multiple copies of genes related to the CzcC family of heavy-metal RND efflux outer membrane proteins suggest resistance to cadmium, cobalt, and zinc, among others (14). The MexE-MexF-OprN multidrug efflux system was also identified in the genome, which likely contributes to this strain’s potential to tolerate the stress of heavy metals and multiple antibiotics (15, 16).

### Accession number(s).

This draft genome sequence has been deposited in GenBank under the accession number CP018743.
